# The lactylation modification of proteins plays a critical role in tumor progression

**DOI:** 10.3389/fonc.2025.1530567

**Published:** 2025-03-19

**Authors:** Dehao Yi, Ke Zhou, Yinlong Pan, Huazhong Cai, Pan Huang

**Affiliations:** ^1^ Department of Emergency, Affiliated Hospital of Jiangsu University, Zhenjiang, China; ^2^ School of Medicine, Jiangsu University, Zhenjiang, China

**Keywords:** lactylation modification, tumor, Angiogenesis, Drug Resistance, TME Warburg effect and lactylation

## Abstract

Lactylation modifications have been shown to be a novel type of protein post-translational modifications (PTMs), providing a new perspective for understanding the interaction between cellular metabolic reprogramming and epigenetic regulation. Studies have shown that lactylation plays an important role in the occurrence, development, angiogenesis, invasion and metastasis of tumors. It can not only regulate the phenotypic expression and functional polarization of immune cells, but also participate in the formation of tumor drug resistance through a variety of molecular mechanisms. In this review, we review the latest research progress of lactylation modification in tumors, focusing on its mechanism of action in angiogenesis, immune cell regulation in tumor microenvironment (TME), and tumor drug resistance, aiming to provide a theoretical basis and research ideas for the discovery of new therapeutic targets and methods. Through the in-depth analysis of lactylation modification, it is expected to open up a new research direction for tumor treatment and provide potential strategies for overcoming tumor drug resistance and improving clinical efficacy.

## Warburg effect and lactylation

In normal cells, glucose is converted to pyruvate in the cytoplasm via the glycolytic pathway. When oxygen levels are sufficient, pyruvate enters the mitochondria and is thoroughly oxidized through the tricarboxylic acid cycle (TCA cycle) to produce carbon dioxide and water, and releases a large amount of energy. However, under hypoxic conditions, pyruvate is reduced to lactate under the catalysis of lactate dehydrogenase (LDH). At the beginning of the 20th century, Warburg discovered that tumor cells still take up glucose in large quantities and convert it to lactate even under aerobic conditions, a phenomenon known as aerobic glycolysis, also known as the Warburg effect (1). The Warburg effect not only plays an important role in tumors, but is also strongly associated with a variety of diseases, including sepsis and autoimmune diseases (2). This discovery opens up a new direction for the study of lactate and tumors. Studies have shown that elevated lactate levels are positively correlated with the incidence of distant metastases in primary human tumors (3), and high lactate levels have also been shown to be important predictors of tumor recurrence and reduced patient survival (4).

Lactylation is an emerging post-translational modification of proteins characterized by the attachment of lactate molecules to lysine residues in the form of covalent bonds. Tumor cells can use the large amount of lactate produced by the Warburg effect as a feedstock to induce lactylation of intracellular proteins, but at the same time, in order to prevent excessive intracellular acidification, the lactate in tumor cells will be transported out of the cell via monocarboxylic acid transporter (MCT4), which maintains a low intracellular lactate concentration and allows aerobic glycolysis and lactate production to continue, while increasing the lactate concentration in the extracellular TME (5). Extracellular lactate, on the other hand, induces intracellular lactylation through two main pathways. On the one hand, extracellular lactate enters the cell through monocarboxylic acid transporters such as MCT1 and MCT2 (6), accumulating and increasing intracellular lactate levels, thus providing the necessary raw materials and environmental conditions for protein lactylation. On the other hand, extracellular lactate, as a signaling molecule, activates G protein-coupled receptors (GPCRs) on the cell surface, especially GPR81, which belongs to the G protein-coupled receptor family and mainly functions through the Gi signaling pathway (7). When extracellular lactate activates GPR81, it activates transcription factors through related signaling pathways, such as activating the mitogen-activated protein kinase (MAPK) signaling pathway, which phosphorylates extracellular signal-regulated kinase (ERK) and enters the nucleus, phosphorylates transcription factor Elk-1, etc., thereby promoting the expression of genes related to lactate metabolism and lactylation. This includes increased expression of the monocarboxylic acid transporter (MCT) gene, which promotes the synthesis of MCTs (8), allowing cells to take up more extracellular lactate. Through this direct and indirect dual mechanism, extracellular lactic acid enters the cell, which provides sufficient raw materials for intracellular lactylation and regulates the process of lactylation.

## Lactylation modification

**Figure 1:**
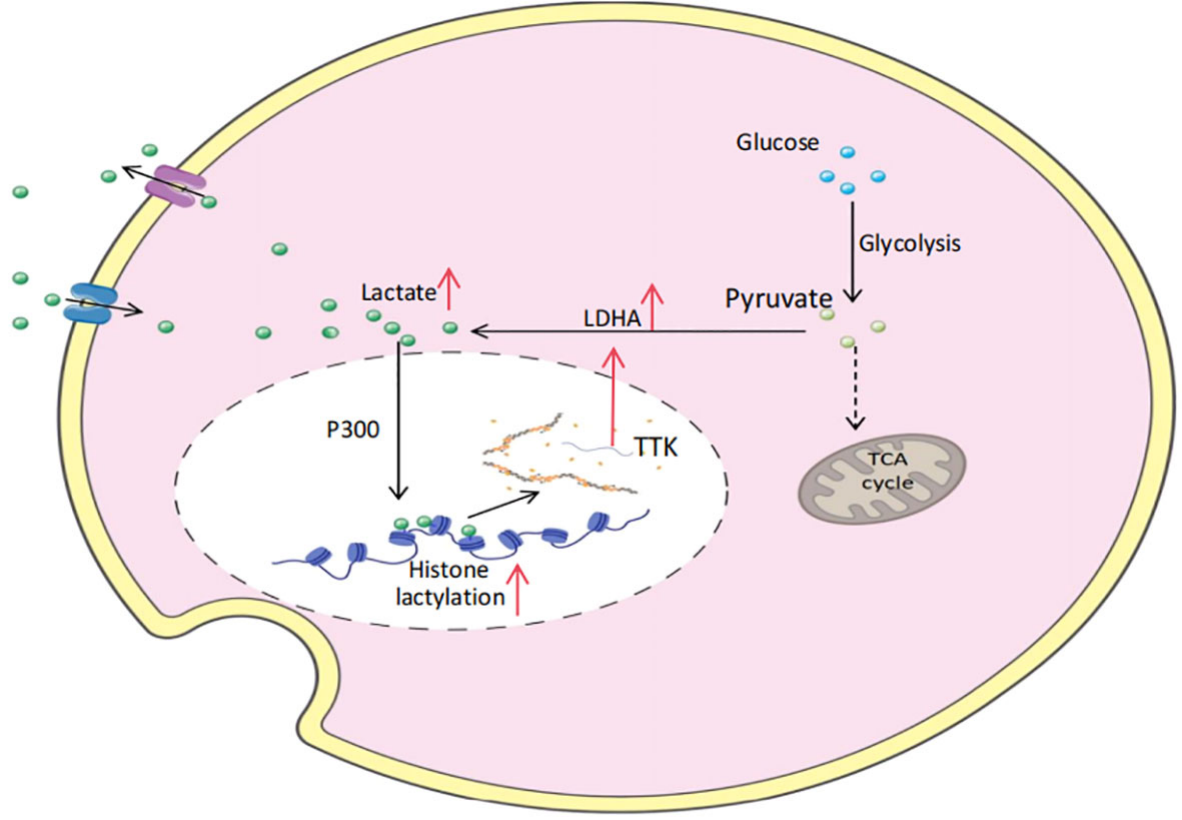
Lactylation positive feedback regulation of pancreatic ductal adenocarcinoma (PDAC).

In 2019, Zhang et al. identified histone lysine lactylation (Kla) as a novel epigenetic modification and demonstrated its direct involvement in the transcriptional regulation of chromatin (9). This modification has been revealed to be a dynamic and reversible process, co-regulated by a specific lactate-based transferase (“writer”) and a delactylating enzyme (“eraser”), which add or remove lactate groups from modified lysine residues, respectively. According to their study, the acetyltransferase p300 is proposed to function as a lactate transferase, mediating histone lactylation by utilizing lactyl-CoA as a lactate donor (10). In a separate study, Ju et al. discovered that histone H3 lactylation could not be effectively catalyzed at physiological lactyl-CoA concentrations of 100 μM. However, Alanyl-tRNA synthetase (AARS1) was found to efficiently catalyze histone H3 lactylation in the presence of physiological concentrations of ATP and lactate. Furthermore, the knockout of AARS1 in the human gastric cancer (GC) cell line HGC27 significantly reduced the levels of histone H3 K18 lactylation, confirming that AARS1 directly utilizes lactic acid and ATP to catalyze protein lactylation (11). Intriguingly, AARS1 not only functions as a lactate transferase but also serves as an intracellular lactate sensor (12). The processes mediating lysine deacetylation primarily involve two enzyme families: histone deacetylases (HDAC1-3) and sirtuins (SIRT1-3). *In vitro* studies have demonstrated that HDAC1-3 significantly reduce the levels of H3K18la and H4K5la, while SIRT1-3 exhibit a milder effect on these modifications. Among these, HDAC3 has been identified as the most effective eraser of both L- and D-lactate lysine modifications *in vitro* (13). Through the coordinated regulation of lactyltransferase “writers” and delactylase “erasers,” histone lactylation influences the initiation and progression of tumors. For instance, in pancreatic ductal adenocarcinoma (PDAC), p300 and HDAC2 have been proposed as potential writer and eraser proteins of histone lactylation, exacerbating PDAC dysfunction through a positive feedback loop involving lactylation (14) (**Figure 1**, **Table 1**). However, the presence of additional cofactors *in vivo* remains to be conclusively demonstrated.

**Table 1:** Functions of H3K9 and H3K18 lactylation on tumor cells.

Location	Downstream Target	Mechanism	Function	Tumor
H3K9	IL-11	By promoting immune checkpoint expression through the JAK2/STAT3 signaling pathway in CD8 T cells+	Promoting CD8 T cell dysfunction and tumor progression	Oral and Pharyngeal Squamous Cell Carcinoma (HNSCC) (31)
	Enhance Laminin Subunit Gamma 2(LAMC2) Transcription	LAMC2 activates the PI3K/Akt signaling pathway to affect VEGFA expression	LAMC2 promotes the migration and invasion of ESCC *in vitro* and enhances its metastasis *in vivo*	Esophageal Squamous Cell Carcinoma (ESCC) (32)
	Inhibit MutL Homolog 1 is a key factor of MMR (MLH1) expression	LUC7 Like 2, Pre-mRNA Splicing Factor(LUC7L2) promotes the retention of intron 7 in the MLH1 transcript, leading to NMD-dependent degradation and suppression of MLH1 expression, and inhibiting the (mismatch repair)MMR pathway.	Promote Temozolomide (TMZ) Resistance	Glioblastoma Multiforme (GBM) (33)
H3K18	Transcription Activation of POM121 Membrane Channel Protein	H3K18la directly activates the transcription of pore membrane protein 121 (POM121), thereby enhancing MYC nuclear transport and directly binding to the CD274 promoter to induce PD-L1 expression.	Support for Immunosuppression in Non-Small Cell Lung Cancer	Non-small Cell Lung Cancer (NSCLC) (34)
	Activate the transcription of TTK protein kinase (TTK) and BUB1 mitotic checkpoint serine/ threonine kinase B (BUB1B) in PDAC	TTK and BUB1B deficiencies suppress the malignant tumor of PDAC, while TTK overexpression partially counteracts the anticancer effect of lactylation inhibition	TTK and BUB1B are involved in the progression of pancreatic ductal adenocarcinoma (PDAC), and TTK is involved in the progression of PDAC malignant tumors mediated by H3K18la.	Pancreatic Ductal Adenocarcinoma (PDAC) (14)
	Increased Vascular Cell Adhesion Molecule 1 (VCAM1) transcription	VCAM1 enhances AKT-mTOR-mediated D-X-C Motif Chemokine Ligand 1(CXCL1) expression, promoting the recruitment of human bone marrow-derived mesenchymal stem cells (hMSCs)	Promoting tumor cell proliferation, Epithelial-Mesenchymal Transition(EMT) transformation, and tumor metastasis	Gastric Cancer GC (35)
	Inhibition of Retinoic Acid Receptor γ (RARγ) gene transcription in the chromatin	RARγ regulates TRAF6-IL-6-STAT3 signaling to link inflammation and tumor development	Promoting tumor growth in the TME	Colorectal Tumor (36)
	Enhance YTH N6-methyladenosine RNA-binding protein 2 (YTHDF2) Expression	YTHDF2 recognizes m6A-modified PER1(tumor suppressor genes) and TP53 mRNA and promotes their degradation	Accelerating the Onset of Ocular Melanoma Tumors	Melanoma (15)
	Activated the transcription of rubicon like autophagy enhancer (RUBCNL) in colorectal cancer	RUBCNL mediates the recruitment and functional activation of the PtdIns3K complex to promote the maturation of autophagosomes	The role of tumor occurrence and progression	Colorectal Cancer (CRC) (37)
	Histone H3K18la lactylation can promote the transcription of Ubiquitin Specific Peptidase 39 (USP39)	USP39 stabilizes the PGK1 protein through proteasome-dependent deubiquitination, thereby activating the PI3K/AKT/HIF-1α pathway	Promoting the Progression of Endometrial Cancer	Endometrial Cancer (38)
	Inducing transcription of downstream targets including LDHA itself	Potassium Two Pore Domain Channel Subfamily K Member 1 (KCNK1) promotes lactylation of H3K18 in breast cancer cells through LDHA, and lactylation of H3K18 induces the transcription of downstream targets, including LDHA itself, leading to a positive feedback loop	Induce the proliferation, invasion, and metastasis of breast cancer cells	Breast cancer (39)
	Regulate the expression of Tumor Necrosis Factor Superfamily Member 9 (TNFSF9)	Hypoxia induces the accumulation of LA in glioma cells through glycolysis, which is then absorbed by macrophages and leads to their M2 polarization via the MCT-1/H3K18La/TNFSF9 axis	Prominently promotes the malignant progression of glioma cells	Glioma (40)

In conclusion, lactylation represents an emerging post-translational modification (PTM). Current knowledge regarding lactylation modification sites, regulatory mechanisms, and associated proteins remains limited, necessitating further investigation to elucidate its full biological significance.

## Histone and non-histone proteins lactylation in tumors

As an emerging post-translational modification (PTM) mechanism, protein lactylation modification (Kla) has become a research hotspot in the field of life sciences, particularly in tumor biology. Recent studies have demonstrated that protein lactylation is widely prevalent in various tumor cells, encompassing both histone and non-histone lactylation. This modification significantly influences tumor cell proliferation, differentiation, and adaptation to the microenvironment by regulating intracellular gene expression, signal transduction, and protein function. Yu et al. were the first to reveal the critical role of histone lactylation in driving oncogene expression and promoting tumorigenesis (15). For instance, lactylation at H4K12 inhibits the transcriptional activity of the Schlafen 5 (SLFN5) promoter, impairing SLFN5’s ability to induce apoptosis in tumor cells and thereby accelerating the progression of triple-negative breast cancer (TNBC) (16). Additionally, histone lactylation is involved in the regulation of cell signaling pathways. For example, Xie et al. demonstrated that circXRN2 activates the Hippo signaling pathway by binding to the LAST1 protein, protecting it from SPOP-mediated ubiquitination and degradation (17). Further research progress on histone lactylation is summarized in detail in **Table 1**.

Non-histone lactylation also plays a pivotal role in tumorigenesis and progression. Yang et al. identified 9,275 lactylation sites, of which 9,256 were located on non-histone proteins, highlighting the significant contribution of non-histone lactylation to tumor proliferation and migration through genomic lactylation analysis of hepatitis-associated hepatocellular carcinoma (HCC) (18). For example, discoidin, CUB, and LCCL domain-containing protein 1 (DCBLD1), an oncogene involved in multiple regulatory mechanisms of tumor progression (19),undergoes lactylation at lysine K172. This modification promotes the proliferation and metastasis of cervical cancer cells by activating the pentose phosphate pathway (20). Furthermore, non-histone lactylation can modulate tumor progression by regulating protein function. In clinical pancreatic cancer samples, lactylation levels of the transcription factor TFEB are significantly elevated. Lactylation at K91 prevents TFEB’s interaction with the E3 ubiquitin ligase WWP2, thereby inhibiting its ubiquitination and proteasomal degradation and ultimately enhancing TFEB activity (21). Nucleolin (NCL), the most abundant RNA-binding protein in the nucleolus, can be lactylated by the acyltransferase p300 under highly active glycolytic conditions. This modification significantly promotes the proliferation and invasion of intrahepatic cholangiocarcinoma (iCCA) cells. Further studies revealed that NCL lactylation upregulates the expression of MAPK-activated death domain protein (MADD) by modulating RNA splicing processes, thereby preventing premature translation termination. The lactylation of NCL and its regulation of MADD expression not only enhance the growth of xenograft tumors but also correlate closely with the overall survival of iCCA patients (22).

These studies collectively suggest that lactylation of both histones and non-histones plays a crucial role in tumor development and progression. These modifications significantly impact tumor cell proliferation, apoptosis, and migration by regulating gene expression, signaling pathways, metabolic processes, and protein function. Moreover, the interplay between the lactate metabolome and the epigenome may provide new directions for epigenetic regulation and tumor therapy. Although previous studies have preliminarily elucidated the importance of lactylation in tumors, the specific molecular mechanisms remain relatively limited. In particular, further research is needed to accurately identify lactylation sites, systematically explore downstream targets, and elucidate the functional significance of known lactylation sites. This field holds great research potential and is expected to provide novel biomarkers and therapeutic targets for the early diagnosis and precision treatment of tumors through deeper mechanistic exploration in the future.

## Lactylation modification in physiological state

As a donor, the concentration of lactate is directly and positively correlated with the level of lactylation modification (Kla). However, whether the large amounts of lactic acid produced by strenuous exercise lead to lactylation modification in normal tissues and whether this lactylation has a similar pro-cancer effect as that observed in tumor cells remain questions worthy of further exploration. Some researchers have investigated these questions using high-intensity interval training (HIIT) models. HIIT represents the primary metabolic scenario for lactate production. Studies have found that a single session of HIIT induces Kla modification in tissues with active lactate uptake and metabolism, such as inguinal white adipose tissue (iWAT), brown adipose tissue (BAT), soleus muscle, and liver. Kla levels peak 24 hours after HIIT and return to a steady state by 72 hours. Further analysis suggests that Kla modification in iWAT may affect *de novo* fat synthesis by regulating pathways related to glycolipid metabolism. Therefore, it is speculated that changes in energy expenditure, lipolytic effects, and metabolic properties during the recovery period after HIIT may be closely related to the regulation of Kla in iWAT (23).

Moreover, exercise-induced lactylation modifications exhibit tissue specificity, occurring primarily in metabolically active tissues (e.g., soleus muscle, adipose tissue, and liver), which are also the primary sites for lactate uptake, clearance, and utilization. Under physiological conditions, lactate concentrations are typically maintained between 1.5 and 3 mM to ensure stable serum lactate levels and to avoid lactic acidosis (4). However, in cancer patients, lactate concentrations can be significantly elevated to 10–30 mM and may even reach abnormally high levels (up to 50 mM) in the tumor core region (24). As early as the 20th century, Warburg observed a 70-fold increase in lactate accumulation when cancer cells were cultured in 13 mM glucose (1). According to his calculations, tumor cells have an arterial glucose uptake rate of approximately 47–70%, compared to 2–18% in normal tissues, and convert about 66% of the glucose they uptake into lactate (25). In tumors with active glycolysis, lactate levels in cancer cells can increase significantly to 40 times the normal level, resulting in a decrease in the pH of the tumor microenvironment to 5.6, although it typically ranges between 6.0 and 7.0 (26). This persistently low pH environment has been shown to significantly impair the function of CD8+ and CD4+ T lymphocytes, including their cytotoxicity, chemotaxis, and proliferative capacity (27). Since lactate concentration is directly related to Kla levels, elevated lactate concentrations in tumor cells can further induce a range of lactylation modifications in proteins, thereby promoting tumorigenesis and progression.

Lactylation is not an inherent pathological mechanism, and protein lactylation exists in some normal cells as well, such as in embryonic stem cells, where lactate can enhance H3K18la, thereby promoting the transcriptional elongation of downstream target genes (28). Kla is also widely present in human lung tissue in a normal physiological state and may be involved in various biological processes, such as RNA splicing, actin filament organization, and neutrophil degranulation (29). In addition, Hagihara et al. identified 63 candidate lactylation proteins in the brain. Their experiments also showed that the addition of lactate and the induction of neuronal excitation can stimulate the increase in Kla levels, and pointed out that stress preferentially increases the Kla level of histone H1 (30).

## Lactylation promotes the formation of tumor blood vessels

Tumors are rapidly proliferating, metabolically active, and highly adaptable biological tissues that require significantly more nutrients than normal cells. To sustain their growth, invasion, and metastasis, tumors must form new vascular networks through angiogenesis. This process constitutes a complex and dynamic system, finely regulated by a balance of pro-angiogenic and anti-angiogenic factors. Among the hypoxia-inducible factor (HIF) family, HIF-1 plays a pivotal role as a key regulator of angiogenesis under hypoxic conditions. HIF-1 is a heterodimer composed of a constitutively expressed HIF-1β subunit and an oxygen-sensitive HIF-1α subunit (encoded by the HIF1A gene). HIF-1α acts as a transcriptional enhancer of vascular endothelial growth factor A (VEGFA) and directly regulates VEGFA expression, which is the primary driver of angiogenesis (41, 42). Further studies have demonstrated that H3K9 lactylation in endothelial cells promotes angiogenesis by enhancing the expression of pro-angiogenic protein-related genes, including NECTIN1 (43), TGFBR2 (44), ABL1 (45), PTGFR (46), LAMA4 (47), CLASP2 (48), PRCP (49), and EGFR (50), in response to VEGF stimulation. Additionally, a positive feedback regulatory loop has been identified: VEGF stimulation not only increases H3K9 lactylation levels but also suppresses the expression of the histone deacetylase HDAC2. The downregulation of HDAC2 further enhances H3K9 lactylation, thereby amplifying the expression of angiogenesis-related genes. Overexpression of HDAC2 in endothelial cells disrupts this VEGF/H3K9la/HDAC2 feedback loop and significantly inhibits angiogenesis (51).In prostate cancer (PCa), KIAA1199 (also known as cell migration-inducing protein/CEMIP) promotes tumor progression by regulating lactate metabolism and VEGFA expression (52). Lou and colleagues found that KIAA1199 is highly expressed in prostate cancer tissues and positively correlates with HIF-1α levels and angiogenesis. HIF-1α-induced lactylation further enhances KIAA1199 expression, thereby increasing the angiogenic capacity of prostate cancer. Silencing KIAA1199 disrupts hyaluronic acid (HA)-mediated VEGFA signaling, upregulates Sema3A expression, and reduces the levels of VE-cadherin and p-EphA2, ultimately inhibiting PCa angiogenesis (53). Based on these findings, Yu and colleagues discovered that evodiamine inhibits Sema3A-mediated angiogenesis and PD-L1 expression in PCa cells by inducing ferroptosis and interfering with HIF1A-mediated histone lactylation (54). Microglia, the resident immune cells of the retina, play a critical role in angiogenesis and vascular diseases and are among the first immune cells activated under hypoxic conditions (55, 56). During pathological angiogenesis induced by retinal hypoxia, microglia are recruited to the lesion site, where they tightly associate with newly formed blood vessels and exert significant regulatory functions (57). Studies have shown that the lactylation level of the transcription factor YY1 in microglia is markedly upregulated under hypoxic conditions. Hypoxia-induced hyperlactylation of YY1 further activates FGF2 expression, thereby promoting neovascularization (58). Additionally, high expression of BCAM has been associated with increased angiogenesis (59). In oral squamous cell carcinoma (OSCC), elevated levels of lactylation modification (Kla) activate BCAM expression. Gene knockout experiments confirmed that BCAM plays a key role in OSCC angiogenesis. In OSCC models, inhibition of BCAM significantly reduced CD31 immunofluorescence (IF) staining intensity, VEGF expression levels, and vascular density (60).

These studies suggest that lactylation plays a crucial role in neovascularization, offering a novel potential strategy for the treatment of diseases and tumors. Targeting and inhibiting lactylation to block tumor angiogenesis may represent an effective therapeutic approach. However, the mechanisms underlying lactylation and tumor angiogenesis have only been elucidated in a limited number of tumor types, and their specific roles in other cancers remain to be explored. Further investigation into the regulatory networks of lactylation in angiogenesis across different tumors and its interactions with the tumor microenvironment will not only provide a comprehensive understanding of lactylation’s functions but may also offer new theoretical foundations and practical guidance for developing targeted anti-angiogenic therapies based on lactylation modulation.

## Lactylation in immune cells

An essential prerequisite for lactylation is the elevated lactate levels in the tumor microenvironment (TME) (61). This suggests that the overall lactylation modifications within the TME, including in tumor cells and immune cells, may be significantly increased, leading to systemic alterations in the microenvironment. Tumor cells produce large amounts of lactate through metabolic reprogramming, creating a lactate-rich TME that induces protein lactylation in immune cells. This lactylation modification is not a normal physiological response of immune cells but is exploited by tumor cells to alter immune cell functions, promoting tumor growth, proliferation, invasion, and metastasis (62). In essence, tumor cells “hijack” the normal regulatory mechanisms of immune cells, repurposing them to support tumor survival and progression. Tumor cells achieve this by controlling epigenetic modifications and enhancing immunosuppressive signaling pathways.

### Control of epigenetics

Lactate accumulation in the TME remodels immune cell metabolism and perpetuates a cycle of malignant tumor behavior, immune cell reprogramming, and environmental immunosuppression (63). For instance, under metabolic stress, tumor-associated macrophages (TAMs) exhibit increased histone lactylation at the H3K18 site, which reduces their anti-tumor phagocytic activity (64). Other studies have shown that lactate activates CCL18 expression through H3K18 lactylation in M2-like macrophages, increasing CCL18 secretion and promoting tumor growth and metastasis *in vivo* (65). Additionally, tumor-infiltrating myeloid cells (TIMs), key players in tumor immune evasion, are regulated by multiple epigenetic mechanisms. Elevated expression of methyltransferase-like 3 (METTL3) in TIMs is associated with poor prognosis in colon cancer patients. Lactate accumulation in the TME induces H3K18 lactylation, which potently upregulates METTL3 expression in TIMs. METTL3-mediated m6A modification of Jak1 mRNA enhances its translation efficiency via the m6A-YTHDF1 axis, leading to increased STAT3 phosphorylation and subsequent immunosuppressive functions (66).

### Enhanced immunosuppressive signaling

In the TME, lactylation modifications at key signaling sites can amplify immunosuppressive signaling, disrupting normal anti-tumor immune responses. For example, lactate-driven MOESIN lactylation enhances the generation of regulatory T cells (Tregs) in the TME. This modification promotes immunosuppression by strengthening the interaction between MOESIN and TGF-β receptors, thereby activating the SMAD3 signaling pathway (67).

Lactylation modifications in tumor-associated cells also play a critical role in regulating immune cell phenotypes, particularly in promoting the transition to immunosuppressive phenotypes. Macrophages, as central players in the immune response, are broadly categorized into two phenotypes: classically activated M1 macrophages and alternatively activated M2 macrophages. In the TME, macrophages typically exhibit an M2 phenotype characterized by immunosuppressive properties. Recent studies have shown that histone lactylation modifications in TAMs are closely linked to their functional polarization, with higher lactylation levels favoring the M2 phenotype. This phenotypic plasticity enables macrophages to play pivotal roles in immune defense, tissue repair, and immune regulation. Zhang et al. demonstrated experimentally that lactate induces the expression of M2 phenotype-related proteins, such as vascular endothelial growth factor, through histone lactylation, thereby promoting M2-like gene expression during M1 macrophage polarization (9). Furthermore, hypoxic conditions drive macrophage polarization toward the M2 phenotype and promote the progression of multiple solid tumors, although the underlying mechanisms remain incompletely understood. Li et al. revealed in glioma studies that hypoxia-mediated lactate accumulation in tumor cells is taken up by macrophages, which then regulate TNFSF9 expression through the MCT-1/H3K18la signaling pathway, inducing M2 macrophage polarization and facilitating glioma progression (40).

Sun et al. further demonstrated that lactate activates CCL18 expression through H3K18 lactylation in M2 macrophages, increasing its secretion and promoting tumor growth and metastasis (65). In studies on tumor resistance, Cai et al. identified SRSF10 as a key gene associated with anti-PD-1 resistance and the immunosuppressive microenvironment in hepatocellular carcinoma (HCC). SRSF10 regulates tumor cell glycolysis through the transcription factor MYB, leading to lactate overproduction. SRSF10 in tumor cells enhances histone lactylation in macrophages, activating genes such as CD206 and inducing M2 macrophage polarization, thereby promoting the formation of an immunosuppressive TME (68).

These studies provide critical insights into the role of lactylation modifications in tumor immune regulation and lay a theoretical foundation for developing lactylation-based therapeutic strategies for cancer treatment.

## Lymphocytes

In exploring the potential role of histone lactylation in T cells, it was found that extracellular lactate treatment promotes the development of regulatory T cells (Tregs) by increasing H3K18 lactylation levels at the Foxp3 locus in Th17 cells. Further analysis revealed that this treatment elevated H3K18 lactylation levels in several genes associated with T cell receptor signaling pathways, such as the NF-κB and MAPK pathways (42). Additionally, lactate can regulate Treg cell production through lactylation of the Lys72 site in the MOESIN protein. Specifically, lactylation enhances the interaction between MOESIN and transforming growth factor-β (TGF-β) receptor I, activating the downstream SMAD3 signaling pathway. This mechanism regulates Treg cells in the tumor microenvironment (TME), influencing tumorigenesis and the efficacy of anti-tumor therapies (67).The anti-tumor activity of natural killer T (NKT)-like cells is significantly suppressed in the “cold” tumor microenvironment characteristic of malignant pleural effusions (MPE) (69). Further studies demonstrated that a chronic high-lactate environment induces FOXP3 expression in NKT-like cells within MPE. ChIP-qPCR analysis identified lactylation modification sites in the FOXP3 gene promoter region of NKT-like cells. The chronic high-lactate environment significantly increased H3K18 lactylation (H3K18la) levels in these cells, while treatment with the lactate transport inhibitor 7ACC2 reduced H3K18la levels. These findings highlight the critical role of lactylation in regulating NKT-like cell function and the tumor microenvironment (70).In mutant KRAS (KRAS^MUT^) cancers, tumor-specific cytotoxic T lymphocytes (CTLs) exhibit heightened sensitivity to activation-induced cell death (AICD). A lactate/NF-κB/AICD regulatory axis was identified in stage IV colorectal cancer (CRC), where circATXN7—a circular RNA interacting with NF-κB—regulates T cell sensitivity to AICD by inhibiting NF-κB activity. Mechanistically, lactate produced by KRASMUT tumor cells directly activates circATXN7 transcription through histone lactylation. circATXN7 binds to the NF-κB p65 subunit, masking its nuclear localization signal motif and sequestering it in the cytoplasm. Notably, circATXN7 is predominantly expressed in tumor-specific CTLs, and its upregulation is strongly associated with poor clinical outcomes and resistance to immunotherapy (71).In human and mouse CD8+ T cells, the enrichment of H3K18 lactylation (H3K18la) and H3K9 lactylation (H3K9la) is considered a key mechanism for initiating the transcription of critical genes and regulating CD8+ T cell function. Studies have also observed that the distribution patterns of H3K18la and H3K9la in different CD8+ T cell subsets are closely linked to their specific metabolic profiles. Preclinical models have shown that modulating H3K18la and H3K9la levels by targeting metabolic and epigenetic pathways can significantly influence the effector functions of CD8+ T cells, including their anti-tumor immunity (72).

These studies reveal the pivotal role of lactylation modifications in regulating T cell function and immune responses within the tumor microenvironment (TME). This research not only provides a novel theoretical foundation for understanding the epigenetic regulation of TME immune cells but also opens new avenues for developing anti-tumor immunotherapy strategies targeting lactylation modifications. Further translation of these basic research findings into clinical applications holds promise for advancing tumor immunotherapy through innovative interventions and therapeutic strategies.

## Lactylation is associated with tumor drug resistance

Tumor drug resistance refers to the ability of tumor cells to tolerate chemotherapy drugs, which is one of the primary reasons for the failure of tumor chemotherapy. The mechanisms underlying tumor drug resistance are highly complex and multifaceted. Metabolic adaptation and epigenetic remodeling are considered critical hallmarks of cancer that may contribute to acquired drug resistance. Chen et al. discovered that MRE11, a key homologous recombination (HR) repair protein, undergoes lactylation in response to DNA damage, a process dependent on ATM phosphorylation. Lactylation of MRE11 enhances its DNA-binding capacity, facilitating DNA end resection and the HR repair process. In patient-derived xenograft models and organoid models, inhibition of MRE11 lactylation through CBP or LDH downregulation significantly suppressed HR repair and increased tumor cell sensitivity to chemotherapeutic agents. Additionally, a cell-penetrating peptide specifically designed to block MRE11 lactylation was developed, which inhibited HR repair and enhanced cancer cell sensitivity to cisplatin and PARP inhibitors (PARPi) (73).Another study reported that lactate-driven lactylation of NBS1 at lysine 388 (K388) is crucial for the formation of the MRE11-RAD50-NBS1 (MRN) complex and the recruitment of HR repair proteins to DNA double-strand break sites. High levels of NBS1 K388 lactylation were significantly associated with poor prognosis in patients undergoing neoadjuvant chemotherapy. Inhibition of lactate production through LDHA gene knockdown or its inhibitor stearopten reduced NBS1 K388 lactylation, thereby decreasing DNA repair efficiency and overcoming tumor cell resistance to chemotherapy (74). This discovery provides new insights into the functional role of aerobic glycolysis in tumor cells. In non-small cell lung cancer (NSCLC), researchers found that lactate enhances lactylation of apolipoprotein C2 (APOC2) at the K70 site. Lactylated APOC2 forms a complex with lipoprotein lipase (LPL), promoting the hydrolysis of triglycerides (TG) into free fatty acids (FFAs) for cellular metabolism. This process contributes to immunotherapy resistance by enhancing regulatory T cell (Treg) activity. Studies demonstrated that an anti-APOC2 K70-lac antibody significantly increased tumor cell sensitivity to PD-1 treatment by inhibiting APOC2 lactylation (75). In hepatocellular carcinoma (HCC), Lu et al. observed that enhanced glycolysis in multiple lenvatinib-resistant models led to lactate accumulation and lactylation of lysine residues in IGF2BP3. This lactylation modification is essential for the stabilization of PCK2 and NRF2 mRNAs, enhancing their expression. This process reprograms serine metabolism and strengthens the antioxidant defense system. Additionally, alterations in serine metabolism increase the availability of methyl donors such as S-adenosylmethionine (SAM), promoting N6-methyladenosine (m6A) methylation of PCK2 and NRF2 mRNAs. The lactylated IGF2BP3-PCK2-SAM-m6A axis maintains high levels of PCK2 and NRF2, enhancing the antioxidant system and promoting lenvatinib resistance in HCC. Treatment with liposomal siRNAs targeting IGF2BP3 or the glycolysis inhibitor 2-DG restored lenvatinib sensitivity *in vivo* (76).In bladder cancer (BCa), Li et al. revealed that enrichment of H3 lysine 18 lactylation (H3K18la) in promoter regions activates key transcription factors YBX1 and YY1, which play a critical role in promoting cisplatin resistance. H3K18la lactylation was identified as a major driver of cisplatin resistance in BCa. Targeting H3K18la effectively restored cisplatin sensitivity in resistant BCa cells (77). In prostate cancer (PCa), long-term enzalutamide (Enz) treatment upregulated the SLC4A4 gene. Mechanistic studies showed that SLC4A4 mediated lactylation of the p53 protein through the NF-κB/STAT3/SLC4A4 signaling axis, promoting Enz resistance and PCa progression. Knockdown of SLC4A4 effectively overcame Enz resistance in both *in vitro* and *in vivo* models (78). In acute myeloid leukemia (AML), particularly in all-trans retinoic acid (ATRA)-resistant acute promyelocytic leukemia (APL) cells, histone lactylation and METTL3 expression levels were significantly upregulated. METTL3 expression is regulated by histone lactylation and direct lactylation modifications. Overexpression of METTL3 promoted ATRA resistance. The compound 20(S)-ginsenoside Rh2 (GRh2) significantly inhibited METTL3 expression in ATRA-resistant APL cells in a concentration-dependent manner, restoring ATRA sensitivity (79). These studies provide critical insights into the role of lactylation modifications in tumor drug resistance and lay a theoretical foundation for developing lactylation-based therapeutic strategies.

Drug resistance in cancer therapy is a multifaceted issue involving numerous factors and mechanisms. Metabolic adaptation, a hallmark of human tumors, is considered a significant contributor to acquired drug resistance and represents a major bottleneck in the treatment of various cancers. In recent years, the role of lactylation modifications in tumor drug resistance has garnered increasing attention, offering a potential new direction for addressing drug resistance. Studies have demonstrated that targeted inhibition of lactylation can restore tumor cell sensitivity to chemotherapeutic agents in multiple contexts. However, despite the scientific significance and therapeutic potential of this field, the specific molecular mechanisms of lactylation in tumors remain poorly understood, and its regulatory networks across different tumor types and microenvironments are not fully elucidated. Therefore, further exploration of lactylation modifications in tumor drug resistance will not only uncover novel mechanisms of resistance but may also provide a theoretical basis and practical guidance for developing targeted therapies based on lactylation modulation.

## Conclusion

As an emerging epigenetic modification mechanism, lactylation plays a pivotal role in tumor biology. Substantial evidence indicates that both histone lactylation and non-histrate lactylation serve as critical regulators in tumor growth and immune evasion processes. This regulatory mechanism is primarily mediated by specialized enzymes, including “writers” and “erasers,” which modulate gene expression through direct and indirect pathways, thereby influencing tumor cell signaling transduction, transcriptional activity, and diverse metabolic processes. Importantly, lactylation modification exerts significant effects on immune cell functionality within the tumor microenvironment (TME), facilitating the polarization of tumor-associated macrophages (TAMs) while simultaneously suppressing lymphocyte-mediated immune signaling, ultimately contributing to tumor immune escape. Furthermore, this modification mechanism supports tumor progression by enhancing angiogenesis, thereby ensuring adequate nutrient supply for proliferating tumor cells. It should be emphasized that lactylation is not exclusively restricted to tumor cells. Research has demonstrated that lactylation modification also regulates lactate metabolism in normal physiological contexts, particularly in tissues characterized by active lactate uptake and metabolism, such as inguinal white adipose tissue (iWAT), brown adipose tissue (BAT), soleus muscle, and liver. A notable distinction exists between exercise-induced lactate concentrations and those generated by tumor cells through the Warburg effect, with the former being substantially lower than the latter. Although the intricate interaction network between lactylation (Kla) and other post-translational modifications (PTMs) remains to be fully elucidated, the homeostatic regulation of lactylation in biological systems has been established as physiologically significant. These scientific discoveries not only elucidate the complex regulatory networks of lactylation modification in disease pathogenesis and progression but also unveil novel research directions and therapeutic potential for future cancer treatment and disease intervention strategies. Given the current state of research in this field, future investigations should prioritize the comprehensive exploration of lactylation’s molecular mechanisms and its potential clinical applications, thereby advancing both theoretical understanding and practical innovations in related disciplines.
